# Cyclodehydration of 1,4-butanediol over Zr-Al Catalysts: Effect of Reaction Medium

**DOI:** 10.3390/ma12132092

**Published:** 2019-06-28

**Authors:** Kuo-Tseng Li, Kuan-Wen Chen

**Affiliations:** Department of Chemical Engineering, Tunghai University, Taichung 40704, Taiwan

**Keywords:** tetrahydrofuran, 1,4-butanediol, Zr-Al mixed oxides, aqueous-phase cyclodehydration, coordinated water

## Abstract

The conversion of 1,4-butanediol (BDO) to tetrahydrofuran (THF) in aqueous phase is desirable because BDO production technology is shifting to bio-based aqueous fermentation routes. In this study, liquid-phase cyclodehydration of BDO to THF was studied in two reaction media (pure BDO and aqueous BDO feeds) at 200–240 °C using ZrO_2_-Al_2_O_3_ (ZA) mixed oxides, which were made with a co-precipitation method and were characterized with XRD, BET, SEM/EDX, pyridine and n-butylamine adsorptions. The maximum acidity and the largest surface area occurred at Zr/Al atomic ratios of 1/1 (ZA11) and 1/3 (ZA13), respectively. The reaction exhibited pseudo-first-order; aqueous BDO feed had much greater rate constant than pure BDO feed, ascribed to the acidic properties of adsorbed water molecules (coordinated to surface metal cations) for the former case. For pure BDO feed, linear relation was observed between rate constant and catalyst acidity, and ZA11 reached a THF yield of 90.1% at 240 °C. With aqueous BDO feed, rate constant increased linearly with increasing surface area and ZA13 reached a THF yield of 97.1% at 220 °C. The change of optimum catalyst composition with reaction medium suggests that active sites for catalyzing BDO cyclodehydration changed with the reaction environment.

## 1. Introduction

Tetrahydrofuran (abbreviated as THF) is an important fine chemical intermediate and a powerful organic solvent. Most THF production is used to make polytetramethylene glycerol (abbreviated as PTMG, also known as poly-THF), which is used in the production of urethane elastomers, polyurethane fibers (ether-based spandex), and copolyester-ether elastomers. The remaining THF production is used as a solvent for the manufacture of poly (vinyl chloride) cements and coating, precision magnetic tape, a reaction solvent in the production of pharmaceuticals, and other miscellaneous uses [1].

THF is typically produced by cyclodehydration of 1,4-butanediol (abbreviated as BDO, shown in Equation (1)), which includes the removal of a OH group from a BDO molecule and the removal of a proton from the same molecule [2].

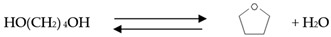
(1)

BDO dehydration is endothermic [2,3] and can be regarded as irreversible [4], especially at high reaction temperature (due to the fact that equilibrium constants for endothermic reactions increase with increasing temperature). It has been reported that BDO equilibrium conversion at 120 °C was 99.75%, and the backward reaction (the reaction between THF and water to form BDO) was not observed [4].

The commercial petroleum-derived BDO technologies include acetylene based process, butadiene-based process, maleic anhydride-based process, propylene-based process via allyl alcohol, and propylene oxide based process. In the commercial processes based on allyl alcohol and propylene oxide, 1,4-BDO is produced by hydrogenation of 4-hydroxyburaldehyde in aqueous solutions [5,6]. For economic reasons, it is better to convert 1,4-BDO to THF in aqueous solutions without water removal because the separation of BDO and water is costly.

BDO production technology is showing signs of transitioning from chemical synthesis routes to optimized bio-based fermentation routes. The emerging BDO technologies include esterification followed by hydrogenolysis of succinic acid (Myriant-Davy Process Technology), fermentation of sugar or biomass (Genomatica, San Diego, CA, USA), and hydrogenation of bio-succinic acid (produced from sugar fermentation, e.g., BioAmber, Montreal, Quebec, Canada) [7]. Most biological processes occur in aqueous solution, therefore, it is desirable to convert 1,4-BDO to THF in aqueous phase.

Reaction (1) can be catalyzed by liquid or solid acids such as strong mineral acids, heteropoly acids, silica gel, alumina, cation exchange resins, ZrO_2_, silica alumina and zeolites [8,9,10]. However, these catalysts have the following disadvantages: mineral acids may corrode equipment and pollute environments [11], heteropoly acids is costly, zeolites deactivate rapidly [12], cation exchange resins exhibited low THF production efficiencies (≦70%) under the rather low temperatures (<120 °C) employed [4,13], vapor phase dehydration of BDO on ZrO_2_ exhibited low THF selectivity (less than 60%) [14].

Compressed water at a temperature above 200 °C possesses very interesting properties [15]. Hunter et al. studied the cyclodehydration of BDO to THF in high-temperature water (200–350 °C) without adding catalyst, THF yield obtained was less than 47% at T ≦ 250 °C [16]. Yang et al. [12] studied cyclodehydration of 1,4-butanediol to THF in near-critical water (260–340 °C) with the addition of three metal sulfates (Fe_2_(SO_4_)_2_, ZnSO_4_, and NaHSO_4_). The maximum THF yield obtained was 59.85 wt % at 320 °C, 120 min with the use of Fe_2_(SO_4_)_2_. It is desirable to decrease the reaction temperature and to increase THF yield for BDO cyclodehydration in aqueous solution using heterogeneous catalysts.

Mixed metal oxides have a wide spectrum of industrial applications [17]. The insertion of foreign metal atoms into the inorganic network of metal oxides is usually used in practice to create active sites with different functionality [18], such as acidic [19,20,21,22,23] or redox functionality [24,25,26,27,28,29,30,31]. ZrO_2_-Al_2_O_3_ mixed oxides have been prepared using sol-gel method [32,33,34,35], electrohydrodynamic atomization [36], co-precipitation method [37,38], laser-splatting [33], plasma spraying [33] and melt extraction [35]. Dabbagh and Zamani used alumina–zirconia mixed oxides (prepared with a sol-gel method) for dehydration of 2-octanol and 1,2-diphenyl-2-propanol to alkenes [32]. No ZrO_2_-Al_2_O_3_ mixed oxides have been used for catalyzing cyclodehydration of BDO to THF.

The major purpose of this work is to decrease the reaction temperature and to increase THF yield for aqueous-phase 1,4-BDO cyclodehydration to form THF by using ZrO_2_-Al_2_O_3_ mixed oxides. The maximum THF yield we obtained for aqueous phase BDO cyclodehydration was 97.1% at 220 °C, which was much better than those reported before. Another purpose of this work is to study the effect of water solvent on the catalytic performances of these catalysts. We found that the optimum Zr-Al catalyst composition changed with reaction medium, which was ascribed to the change of active sites for BDO cyclodehydration.

## 2. Materials and Methods

### 2.1. Catalyst Preparation and Characterization

ZrO_2_-Al_2_O_3_ mixed oxides with Zr/Al atomic ratios of 1/3, 1/1 and 3/1 were prepared by a co-precipitation method. Zirconium tetrachloride (ACROS, 98% purity) and aluminum trichloride hexahydrate (SHOWA, 97%) were dissolved in anhydrous ethanol (ECHO, >99.5% purity). The solution was mixed with aqueous ammonia (SHOWA, 28%). The gel solution was aged at room temperature for 2 h and then the precipitate was filtered and washed with de-ionized water until no chloride ion was left. The precipitate was dried at 110 °C for 12 h, and then heated to 500 °C at a rate of 1 °C /min and kept at 500 °C in air for 4 h. For comparisons, single oxides (ZrO_2_ alone, Al_2_O_3_ alone) were also prepared using the same procedure mentioned above except no aluminum trichloride hexahydrate was added for preparing pure ZrO_2_, and no zirconium tetrachloride was added for preparing pure Al_2_O_3_.

The catalyst specific surface area was determined by nitrogen adsorption with a Micromeritics (Norcross, GA, USA) surface area analyzer (model ASAP 2020). Before nitrogen adsorption, samples were degassed at 150 °C in vacuum for 3 h. Catalyst acidic properties were measured by n-butylamine adsorption. Samples were heated at 150 °C for 12 h and were degassed in vacuum for 3 h before putting in a desiccator with saturated n-butylamine vapor at room temperature for 48 h. The samples were then degassed in vacuum to remove physically adsorbed n-butylamine. Then a TG apparatus (TA Q50, New Castle, DE, USA) was used to measure the weight loss of the adsorbed sample at programmed temperature (with a heating rate of 20 °C/min, started from 30 °C and ended at 550 °C). The presence of Lewis and Bronsted acid sites in the catalyst samples were determined through infrared spectroscopy using pyridine as a probe molecule. Before pyridine adsorption, the samples were heated at 400 °C for 2 h and then cooled to room temperature in a U-shaped tube. Evaporated pyrideine was carried to the samples with flowing nitrogen (250 ml/min) for 30 min. The samples were then analyzed with a FTIR (Shimazu IR-Prestige-21, Kyoto, Japan). Catalyst crystal structure was examined by X-ray diffraction (XRD) crystallography on a Shimadzu XRD-6000 diffractometer with Cu Kα radiation. Quantitative analysis of the elements on the catalyst surface was performed using a field emission scanning electron microscope (JEOL JSM-7000F, Tokyo, Japan) equipped with an Energy Dispersive X-ray Spectrometer (abbreviated as EDS, OXFORD INCA ENERGY 400, Abingdon, Oxfordshire, England).

### 2.2. Reaction Studies

Cyclodehydration of BDO was carried out with a 300 ml stirred reactor (supplied by Parr Instruments Co., Moline, IL, USA). Two types of feed were used: 56 g pure BDO (ACROS, Geel, Belgium; >99% purity) and 2.4 g BDO in 50 ml de-ionized water. The feed and 0.2 g or 0.4 g catalysts prepared above were mixed together and charged into the reactor. The agitator speed was set at 600 rpm, and the reaction mixture was then heated to the desired temperature. The total pressure in the autoclave increased continuously with the progress of reaction due to the conversion of high boiling point BDO (b.p. = 230 °C) to low boiling THF (b.p. = 66 °C). At the end of the reaction, the component compositions were determined with a Shimadzu (Kyoto, Japan) GC-2014 gas chromatography equipped with a 60 m Restek (Bellefonte, PA, USA) Stabilwax-DB capillary column. 1,4-butanediol conversion was defined as the percentage of 1,4-butanediol in the feed that had reacted. THF yield was defined as the moles of THF obtained per mole of 1,4-butanediol in the feed.

## 3. Results and Discussion

### 3.1. BDO Cyclodehydration with Pure BDO Feed

**Figure 1:**
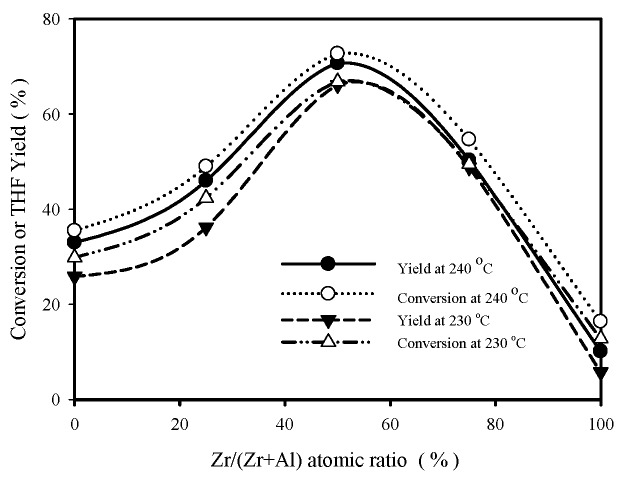
1,4-butanediol (BDO) conversion and THF yield versus Zr/(Zr + Al) molar ratio at 230–240 °C (pure BDO feed). Reaction conditions: 56 g pure BDO, 0.2 g catalyst, t = 3 h.

For pure BDO feed, the effect of catalyst composition (expressed as Zr/(Zr+Al) molar ratio) on BDO conversion and THF yield is shown in Figure 1, using 0.2 g ZrO_2_-Al_2_O_3_ catalysts at 230–240 °C and 3 h reaction time. The catalytic activity decreases in the following order: Zr/Al = 1/1 (denoted as ZA11) > Zr/Al = 3/1(denoted as ZA31) > Zr/Al = 1/3(denoted as ZA13) > Zr/Al = 0/1 (denoted as ZA01) > Zr/Al = 1/0 (denoted as ZA10). That is, ZrO_2_-Al_2_O_3_ binary oxides have significantly better catalytic performances than component oxides and ZA11 catalyst has the highest activity. THF yield is sensitive to the change of reaction temperature, time and catalyst amount. For ZA11 catalyst, THF yield increased from 42.4% at 220 °C to 70.7% at 240 °C (shown in Figure 1, using 0.2 g catalyst and 3 h reaction time), and reached 90.1% with 0.4 g catalyst and 9 h reaction time at 240 °C.

### 3.2. BDO Cyclodehydration Using Aqueous BDO Feed

**Figure 2:**
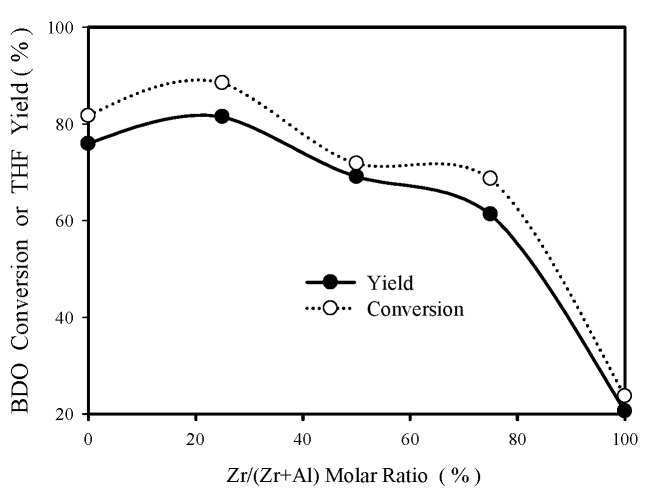
Effect of catalyst composition on THF yield and BDO conversion at 220 °C (aqueous BDO feed). Reaction conditions: 2.4 g BDO in 50 ml water, 0.2 g catalyst, t = 3 h.

For BDO cyclodehydration in aqueous solution, the variation of BDO conversion and THF yield with the change of ZrO_2_-Al_2_O_3_ catalyst composition is shown in Figure 2, using 0.2 g catalyst at 220 °C and 3 h reaction time.

**Figure 3:**
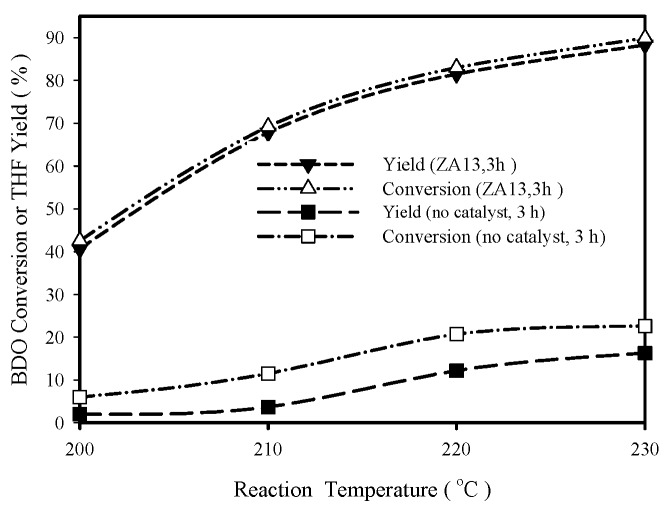
Influence of reaction temperature on tetrahydrofuran (THF) yield (aqueous BDO feed).Reaction conditions: 2.4 g BDO in 50 ml water, 0.2 g catalyst, t = 3 h.

In Figure 2, the activity order is ZA13 > ZA10 > ZA11 > ZA31 > ZA10. That is, ZA13 have the highest THF yield among these Zr-Al oxides. It is interesting to note that the best catalyst changed from ZA11 to ZA13 when the feed was changed from pure BDO to aqueous BDO, which suggests that the active sites for BDO cyclodehydration might be different in these two reaction media. THF yield increased significantly with increasing catalyst amount. For ZA13 at 220 °C and 3 h, THF yield increased from 81.5% to 91.3% when catalyst amount was increased from 0.2 g to 0.4 g. At the absence of catalyst and under the same reaction temperature and reaction time, THF yield was only 12.2%, as shown in Figure 3.

Figure 3 shows the effect of reaction temperature (in a temperature range of 200–230 °C) on ZA13 catalyst performances at 3 h in aqueous solution. Blank run (without catalyst addition) results are also shown in Figure 3 for comparisons. These results indicate that BDO conversion and THF yield increase rapidly with increasing reaction temperature. In the absence of catalyst, THF yields at 3 h are 3.7%, 12.2%, and 16.3% at 210, 220, and 230 °C, respectively. In the presence of 0.2 g ZA13 catalyst, the respective THF yields at 3h are 67.8%, 81.5%, and 88.3%. That is, the addition of 0.2 g ZA13 catalyst greatly improved THF yield.

**Figure 4:**
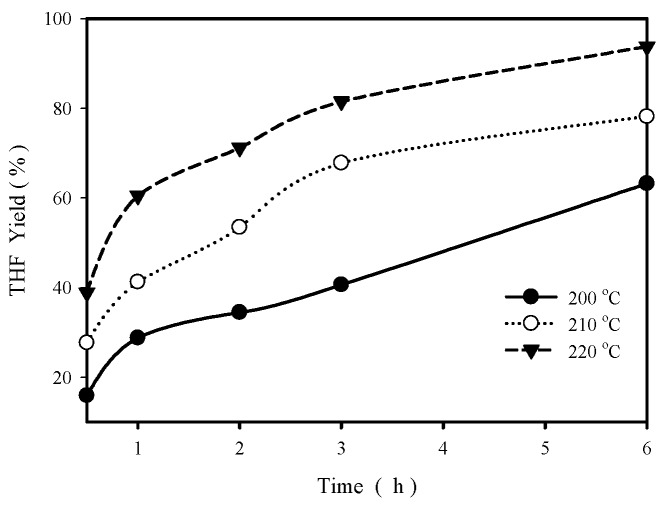
THF yield as a function of reaction time at three different temperatures (200 °C, 210 °C and 220 °C). Reaction conditions: 2.4 g BDO in 50 mL water, 0.2 g catalyst.

Figure 4 shows the effect of reaction time on THF yield in aqueous phase cyclodehydration for ZA13 at three temperatures (200–220 °C), indicating that the increase of reaction time significantly increases THF yield. At 200 °C, THF yield increases from 28.8% at 1 h to 63.2% at 6 h. At 220 °C, THF yield increases from 60.5% at 1 h to 93.8% at 6 h, and reached 97.1% (with a BDO conversion of 98.34%) at 12 h. This should be close to the equilibrium THF yield because of the high BDO conversion and long reaction time.

**Figure 5:**
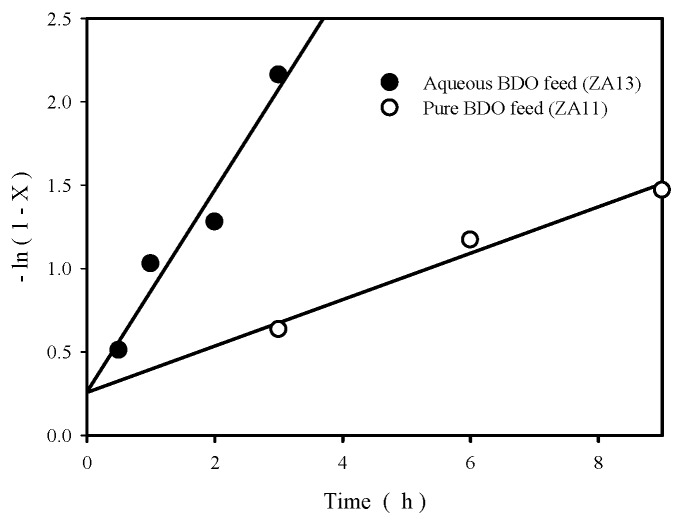
Plots of −ln (1-X) as a function of reaction time (X is BDO conversion) at 220 °C for ZA13 catalyst (with aqueous BDO feed) and for ZA11 catalyst (with pure BDO feed).

Figure 5 compares the plots of −ln(1-X) versus reaction time for ZA13 (with aqueous BDO feed) and ZA11 (pure BDO feed), X is BDO conversion at 220 °C. This kind of plot is often used to test a pseudo-first-order rate law in a batch reactor [39]. Straight lines are obtained in Figure 5, therefore, the rate of BDO disappearance exhibits first-order with respect to BDO concentration. It is observed that the plots show the presence of intercepts (about 0.26) instead of showing zero, as predicted theoretically. The presence of such small intercepts is attributed to BDO conversion occurred during heating period (from room temperature to 220 °C). The rate constants, determined from slopes of the straight lines, are 0.605/h and 0.139/h for aqueous BDO feed (with ZA13 catalyst) and for pure BDO feed (with ZA11 catalyst), respectively. The former has much higher rate constant (4.35 times) than the latter, which are contrary to the results obtained by Vaidya et al. [13] and Baba et al. [9]. Vaidya et al. used a strong acid cation exchange resin to catalyze BDO cyclodehydration in a temperature range of 80–110 °C and found that water inhibited the reaction, which was ascribed to the strong adsorption of water on active sites of resin. Baba and Ono used heteropoly acids to catalyze BDO cyclodehydration at 157 °C and found that the reaction was much slower in water than in 1,4-dioxane, which was ascribed to proton hydrating of the heteropoly acids. The difference between our results and the previous results of Vaidya et al. and Baba et al. might be due to the difference of reaction temperature (water adsorption amount decreases with increasing temperature) and the difference of catalysts used.

For transitional metal oxides in aqueous solution, oxide surface is fully covered with adsorbed water and hydroxyl groups [40]. For molecularly adsorption, a water molecule is coordinated to the surface metal M (M = Al or Zr in this work) via partial transfer of charge from a water molecule to a metal cation (i.e., M **←** OH_2_ transfer), which weaken the OH bond and increase the positive charge on hydrogen. The coordinated water molecules are therefore tend to de-protonate according to Equation (2) [41].

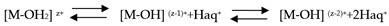
(2)
where z = 3 and 4 for Al and Zr, respectively.

Haq^+^ (abbreviated as H^+^) produced from Equation (2) can catalyze BDO cyclodehydration to THF via one of the following mechanisms:

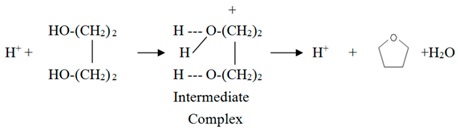
(3)

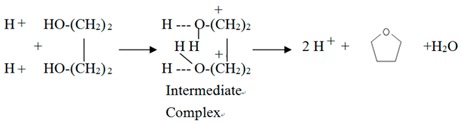
(4)

Equation (3) is a single site mechanism, which has been proposed for BDO cyclodehydration by heteropoly acids [9] and a zeolite (ZSM-5) [8]. Equation (4) is a dual site mechanism, which has been proposed before for BDO cyclodehydration on an acidic ion exchange resin [4]. In Equation (3), the transfer of a proton to oxygen of BDO gives an intermediate complex H_2_OR^+^OH (R = C_4_H_8_), then a H_2_O molecule is lost from the ion, a THF molecule (C_4_H_8_O) is produced and one proton is regenerated. Based on Equation (3), Aghaziarati et al. [8] obtained the following rate equation for BDO (denoted as A) cyclodehydration:

−r_A_ = kC_A_/(1 + K_A_C_A_) (5)

where k is rate constant, K_A_ is adsorption equilibrium constant of A, C_A_ is concentration of A. They found that K_A_ decreased rapidly with increasing reaction temperature with a heat of adsorption = −33.28 kJ/mol. In the reaction condition we used (≧200 °C), K_A_C_A_ is much smaller than 1 because of high reaction temperature, Equation (5) becomes.

−r_A_ = kC_A_ (6)

That is, reaction rate is 1st order with respect to BDO concentration, which is identical to the results we obtained in Figure 5.

### 3.3. Catalyst Characterization Results

**Figure 6:**
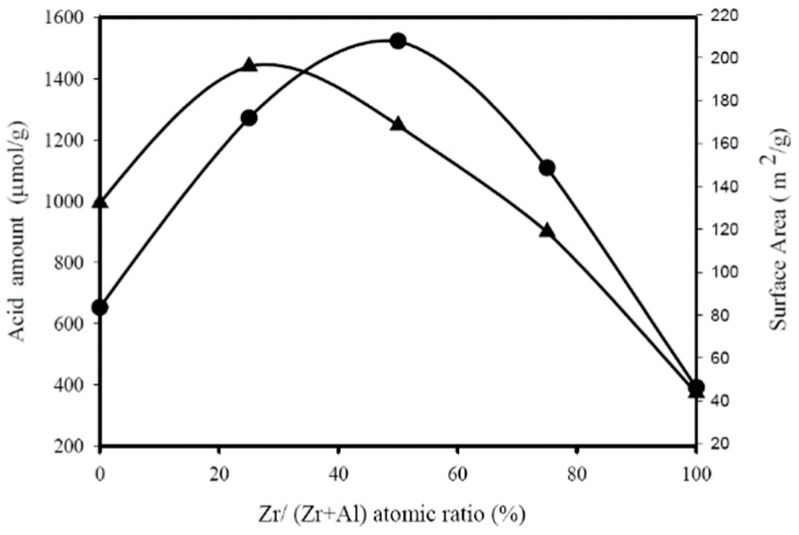
Influence of Zr content on the amounts of acid sites (●, refer to left ordinate) and surface area (▲, refer to right ordinate) of ZrO_2_-Al_2_O_3_ oxides.

Figure 6 shows the effect of catalyst composition on the amounts of acid sites (numerical values are shown on the left ordinate, determined with n-butylamine adsorption) and surface area (numerical values are shown on the right ordinate) for calcined Zr-Al catalysts. Acidity decreases in the following order: ZA11 > ZA13 > ZA31 > ZA01 > ZA10, which is similar to the BDO cyclodehydration activity order obtained with pure BDO feed (ZA11 > ZA31 > ZA13 > ZA01 > ZA10, as shown in Figure 1).

**Figure 7:**
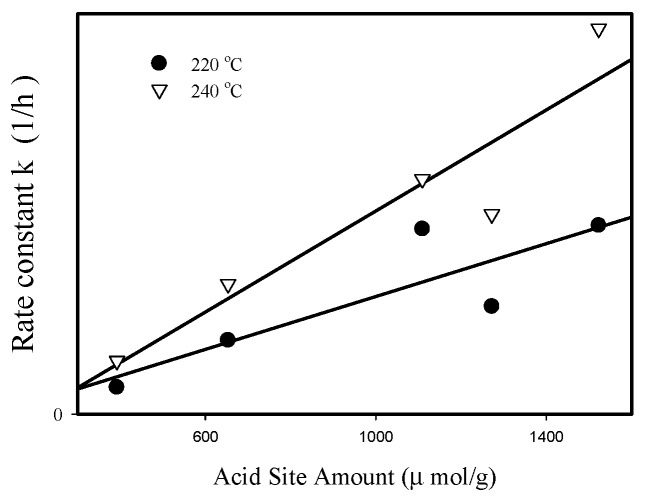
Correlation between first order rate constant k (with pure BDO feed, at 220 °C and 240 °C) and acidity for ZrO_2_-Al_2_O_3_ oxides.

Figure 7 shows that a linear relationship is obtained by plotting first-order rate constants at 220 °C and 240 °C (obtained with pure BDO feed) as a function of catalyst acidity, suggesting that acid sites on Zr-Al catalysts have similar activity for catalyzing BDO cyclodehydration to THF when the feed was pure BDO.

**Figure 8:**
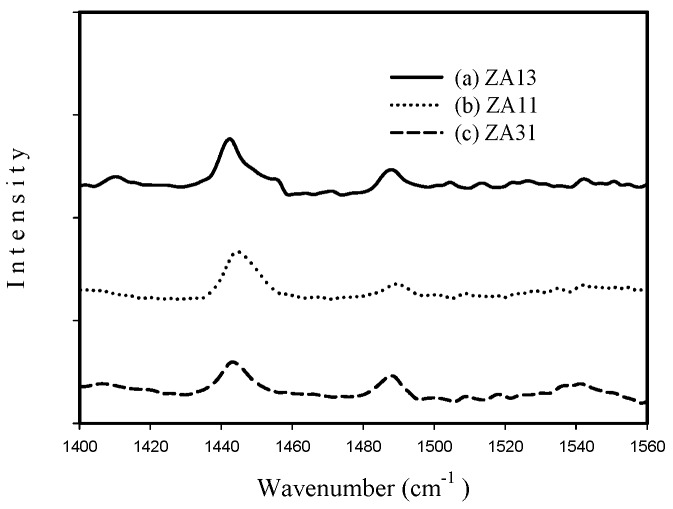
FT-IR spectra in 1400–1560 cm^−1^ spectral region for the fresh (a) ZA13, (b) ZA11, and (c) ZA31 catalysts.

Figure 8 shows the adsorbed pyridine IR spectra of fresh (a) ZA13, (b) ZA11, and (c) ZA31 catalysts. The Bronsted and Lewis acid sites could be distinguished by the bands of chemisorbed pyridinium ion at IR wavenumber ~1540 cm^−1^ and coordinative bonded pyridine at IR wavenumber ~1440 cm^−1^, respectively. The band at IR wavenumber ~1490 cm^−1^ is usually associated with pyridine adsorbed on both Bronsted and Lewis acid sites [42,43].

Figure 8 indicates that the major acid sites existed in the calcined Zr-Al mixed oxides are Lewis acid, because the peak at IR wavenumber ~1440 cm^−1^ is strong (coordinative bonded pyridine due to pyridine on Lewis acid sites) and the peak at IR wavenumber ~1540 cm^−1^ (chemisorbed pyridinium ion due to pyridine on Bronsted acid sites) is weak.

**Figure 9:**
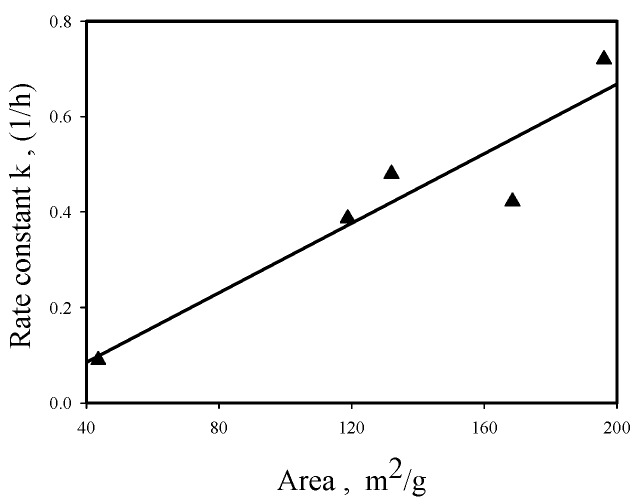
Correlation between first-order rate constant (in aqueous feed, at 220 °C) and surface area for ZrO_2_-Al_2_O_3_ oxides.

The right ordinate of Figure 6 shows the variation of catalyst specific surface area as a function of ZrO_2_-Al_2_O_3_ catalyst composition, indicating that ZA13 and ZA11 mixed oxides have larger surface areas than pure oxides (ZA13 > ZA11 > ZA01 > ZA31 > ZA10). It is interesting to note that the BDO cyclodehydration activity order in aqueous solution (shown in Figure 2) is identical to the surface area order shown in Figure 6. A linear relationship is obtained by plotting the first-order rate constant (obtained with aqueous BDO feed) versus surface area for Zr-Al catalysts, as shown in Figure 9, suggesting that the amount of Haq^+^ produced from Equation (2) is proportional to the catalyst surface area.

For ZrO_2_-Al_2_O_3_ oxides with aqueous BDO feed, areal reaction rates (calculated from Figure 2 and Figure 6) are in the range of 0.22 ± 0.3 mmol THF/m^2^.h for all catalysts, which are not sensitive to the change of catalyst composition, suggesting that all Haq^+^ produced from Equation (2) have similar activity for catalyzing BDO cyclodehydration to THF in aqueous solution.

**Figure 10:**
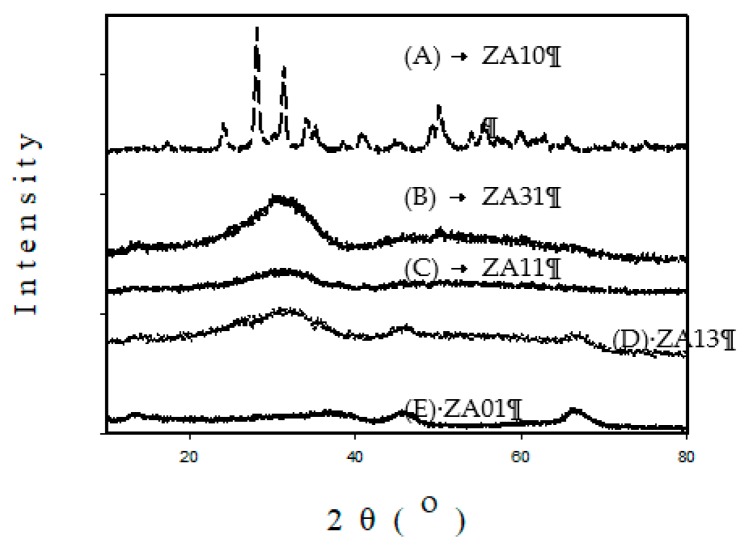
X-ray diffraction (XRD) patterns of (A) ZA10, (B) ZA31, (C) ZA11, (D) ZA13, and (E) ZA01 catalysts.

Figure 10 presents X-ray diffraction (XRD) patterns of (A) ZA10, (B) ZA31, (C) ZA11, (D) ZA13, and (E) ZA01 catalysts. ZrO_2_ is crystalline with high intensity peaks (profile A), indicating that monoclinic phase predominates (peaks at 2θ = 26°, 29°, 31°, 40°, 50°, 53° and 55°) with tetragonal phase present in small amount (peaks at 2θ = 30°, 35° and 62°). It has been reported that the mixture of tetragonal and monoclinic phases has been formed after calcinations of originally amorphous hydrous zirconia at 450 °C [44]. ZA31 and ZA11 are amorphous solids and the broadened peak at 2θ ~30° (profiles B and C) was considered as an appearance of amorphous zirconia [45]. The change from crystalline ZrO_2_ to amorphous ZA31 and ZA11 mixed oxides results in increasing surface area and acid site amount (shown in Figure 6), and increasing activity for BDO cyclodehydration to THF (shown in Figure 1 and Figure 2). ZA13 and ZA01 have two peaks at 2θ ~46° and 66.5° (profiles D and E of Figure 10), which is the semicrystalline γ-alumina phase [46].

EDS analyses indicated that surface Zr/Al atomic ratios for ZA13, ZA11 and ZA31 were 1/3.7, 1/1.8 and 3.3/1, respectively. The measured surface compositions were slightly different from precursor Zr/Al atomic ratios (1/3, 1/1, and 3/1) used for the preparation of these mixed oxides, which might be due to the solubility difference of the compounds involved in the catalyst precipitation process [40].

**Figure 11:**
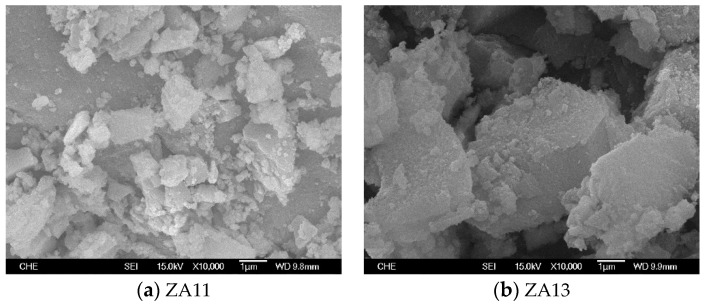
Scanning electron microscopy (SEM) images of (**a**) ZA11 and (**b**) ZA13 catalysts.

Figure 11 presents scanning electron microscopy (SEM) images of (a) ZA11 and (b) ZA13 with a magnification of 10,000, indicating that ZA13 particle size is in the range of 2.5–5 μm, which is significantly larger than that of ZA11 particles (< 2.5 μm). The particle size difference might be due to the fact that ZA13 is partly crystalline solid and ZA11 is amorphous solid, as shown in Figure 10.

## 4. Conclusions

ZrO_2_-Al_2_O_3_ (ZA) mixed oxides with different atomic ratios were prepared with a co-precipitation method. These catalysts were used to catalyze the 1,4-butanediol (BDO) cyclodehydration to produce tetrahydrofuran (THF) with pure BDO feed and with aqueous BDO feed. When the feed was pure BDO, a linear relationship between THF yield and acidity was observed, ZA11 showed the best catalytic activity and reached a THF yield of 90.1% at 240 °C. When the feed was aqueous BDO solution, a linear relationship between THF yield and surface area was observed, ZA13 exhibited the best catalytic activity and reached a THF yield of 97.1% at 220 °C. The pseudo-first-order rate constant obtained with aqueous BDO feed was much greater than that obtained with pure BDO feed, which was ascribed to the protons produced from the coordinated water molecules on the Zr-Al catalyst surface in aqueous solution. ZA11 and ZA13 mixed oxides can reduce commercial THF production cost because they exhibited significant better THF yield than single oxide catalysts under identical reaction conditions.
